# Polyphosphate from Lactic Acid Bacteria: A Functional Molecule for Food and Health Applications

**DOI:** 10.3390/foods14132211

**Published:** 2025-06-23

**Authors:** Daniela Corrales, Cristina Alcántara, Vicente Monedero, Manuel Zúñiga

**Affiliations:** Lactic Acid Bacteria and Probiotics Laboratory, Instituto de Agroquímica y Tecnología de Alimentos (IATA-CSIC), C/Catedràtic Agustín Escardino 7, 46980 Paterna, Spain; daniela.corrales@csic.es (D.C.); crisalba@iata.csic.es (C.A.); btcmon@iata.csic.es (V.M.)

**Keywords:** lactic acid bacteria, *Lactobacillaceae*, polyphosphate, polyphosphatase, probiotic

## Abstract

The linear polymer polyphosphate (polyP) is found across all three domains of life and fulfills diverse physiological functions, including phosphorus storage, chaperone activity, and stress tolerance. In bacteria, polyP synthesis is catalyzed by polyphosphate kinase (Ppk), whereas its degradation is carried out by exopolyphosphatases (Ppx). Intracellular polyP levels are determined by the balance between these opposing enzymatic activities, although the regulatory mechanisms governing this balance remain incompletely understood. In higher eukaryotes, polyP participates in diverse physiological processes from cell signaling to blood clotting. In relation to this, polyP from *Levilactobacillus brevis* has been identified as a protective factor against intestinal damage in a mouse model of acute colitis. Subsequent evidence has confirmed that polyP can confer beneficial effects on human intestinal health, prompting an increased interest in the production of polyP by probiotic lactic acid bacteria. Furthermore, polyP is extensively used in the food industry to enhance food quality, preservation, and nutritional value. This review summarizes the current knowledge on polyP metabolism in these bacteria and explores its functional properties and potential applications.

## 1. Introduction

**Figure 1:**
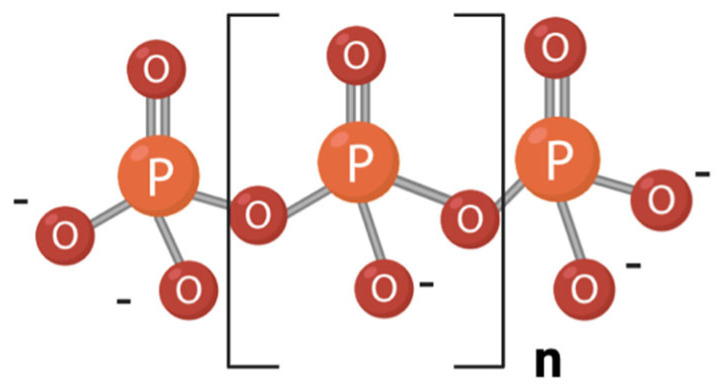
Structure of a linear polyphosphate (polyP) molecule. The number of phosphate residues (n) can range from tens to hundreds.

Polyphosphate (polyP) is a linear polymer composed of phosphoryl monomers linked by high-energy phosphoanhydride bonds (Figure 1). PolyP chains range in length from three to several hundred residues. Although considered to exist almost exclusively as a linear polymer within organisms, recent evidence suggests that alternative configurations, such as branched polyP, may be more stable in aqueous media than previously thought [1,2]. PolyP can also be produced abiotically in environments such as volcanoes [3] and it is thought to have been present on prebiotic Earth, contributing to the conditions for the emergence of life. It may have been incorporated into prebiotic vesicles, serving as a source of inorganic phosphate (Pi) and high-energy bonds and potentially acting as a precursor to ATP [4]. This molecule is found in every type of cell, including bacteria, archaea, fungi, protozoa, plants, and animals [4,5,6].

In bacteria, polyP plays multiple roles, including phosphate and energy storage, survival under starvation, stress response, regulation of cell motility, biofilm formation, and virulence [4,5]. Additionally, polyP mitigates stress-induced DNA damage [7] and influences enzyme activity [2,5,8,9]. It can also form a wide variety of complexes with inorganic ions, proteins, and nucleic acids [2]. PolyP was first noticed through the study of characteristic metachromatic inclusions, originally described over a century ago in *Spirillum volutans* and classically named volutin granules [10]. Electron diffraction studies have shown that these structures are amorphous, and elemental analysis with energy-dispersive X-ray spectroscopy has demonstrated that they are enriched in divalent cations Mg^2+^ and Ca^2+^ [2].

PolyP has attracted significant interest in biotechnological applications. The removal of phosphorus from wastewater by polyP-synthesizing bacteria, although still minor in wastewater treatment plants, has shown promising results and offers several advantages over chemical methods, such as eliminating the need for chemical precipitants and reducing sludge volumes [11,12]. PolyP-accumulating microalgae and cyanobacteria have also been proposed as a means to mitigate water eutrophication and phosphorus cycling for agriculture [13]. Owing to its highly variable properties, depending on chain length, cation composition, solvent, etc., polyP has been widely used in different industrial applications, such as fertilizer, fire retardant, or additive for different materials [14]. PolyP has also attracted interest for the development of biomedical materials in regenerative medicine [15,16]. In the food industry, polyP is extensively used to enhance food quality, preservation, and nutritional value. It improves water-holding capacity, stabilizes emulsions, and fortifies foods with essential minerals [17,18]. Furthermore, in recent years, several studies have shown that polyP produced by some bacteria can contribute to the maintenance of intestinal homeostasis. PolyP was initially proposed as the agent responsible for the probiotic activity of *Levilactobacillus brevis* SBC8803 [19]. This finding has led to renewed interest in polyP synthesis by lactic acid bacteria (LAB).

LAB were first defined by Orla-Jensen as a group of Gram-positive, non-sporulating, non-motile bacteria with coccoid or rod forms and the ability to catabolize sugars, mainly to lactic acid [20]. This classification encompassed a heterogeneous group, including bifidobacteria and lactobacilli, among others. Advances in molecular phylogenetic analyses have since clarified the taxonomic status of the bacterial groups formerly included in LAB. Today, the term LAB specifically refers to organisms belonging to the phylum *Bacillota*, class *Bacilli*, and order *Lactobacillales*. Phylogenetic studies indicate that *Lactobacillales* constitutes a monophyletic group [21,22], comprising the families *Aerocococcaceae*, *Carnobacteriaceae*, *Enterocococcaceae*, *Lactobacillaceae,* and *Streptococcaceae*. Many LAB species are considered Generally Recognized as Safe (GRAS) by the US FDA or QPS (Qualified Presumption of Safety), according to European legislation [23], due to their long-standing use in the production of fermented foods and their natural presence as commensals in the human gastrointestinal tract. Furthermore, some of them are considered as health-promoting bacteria.

Given the functional properties of polyP, and the widespread use of LAB in the food industry and probiotic formulations, understanding how LAB regulate polyP synthesis and degradation could help to enhance their functional potential and optimize their application in fermented products and nutritional supplements. Investigating the metabolic pathways involved in polyP metabolism in LAB not only deepens our fundamental understanding of LAB physiology, but also opens new opportunities for biotechnological and health-related applications. This review aims to provide a comprehensive overview of polyP synthesis in food-associated LAB, focusing on the key enzymes involved, their physiological roles and potential health applications. The use of polyP in food technology will not be addressed in this review, as it has been recently revised [18].

## 2. Synthesis of Polyphosphate in LAB

The main enzymes in bacteria involved in polyP synthesis are polyP kinases (Ppk), which, based on their amino acid sequences and kinetic properties, are classified into two families: Ppk1 (EC 2.7.4.1) and Ppk2 (EC 2.7.4.34). The two enzymes share no significant sequence similarity and possess different structural folds [24]. Conversely, exopolyphosphatases (Ppx) hydrolyze polyP, releasing the terminal Pi. On the basis of their primary structures, they are divided into two types, Ppx1 and Ppx2, respectively. Ppx1 consists of a N-terminal domain belonging to the acetate and sugar kinase/hsp70/actin superfamily (ASKHA) and a C-terminal domain which is responsible for the processivity of the enzyme [25]. Ppx2 enzymes share the N-terminal domain but lack the C-terminal domain of Ppx1. PolyP synthesis in fungi and other unicellular eukaryotes is catalyzed by a completely different pathway, namely, the vacuolar transporter chaperone pathway [26,27].

The presence of polyP in LAB was first reported in the early 1960s for *Lactobacillus casei* (currently *Lacticaseibacillus casei*) [28]. Subsequently, Kakefuda et al. [29] identified polyP granules in the cytoplasm of *Lactobacillus plantarum* (currently *Lactiplantibacillus plantarum*) while studying its membrane ultrastructure. Further investigations described polyP in *Lp. plantarum* as a molecule complexing Mn^2+^ as a counterion [30]. The detection of polyP granules in 60% of *Lactobacillus* strains isolated from mozzarella cheese whey suggested that the ability to synthesize polyP is widespread in lactobacilli [31]. This observation has been supported by additional studies reporting polyP in various other *Lactobacillaceae* species [19,32,33,34]. A survey of available LAB genome sequences revealed that genes involved in polyP synthesis and degradation, namely, *ppk1*, *ppx1*, and *ppx2*, are uncommon in *Aerococcaceae*, *Carnobacteriaceae*, *Enterococcaceae*, and *Streptococcaceae*, whereas they are highly prevalent in *Lactobacillaceae* [34,35]. In contrast, *ppk2* genes are present in far fewer *Lactobacillaceae* species (Table 1) and are not found in the absence of *ppk1*. However, the capacity to synthesize polyP is not universal within *Lactobacillaceae*, as several species of lactobacilli lack the signature gene encoding the enzyme required for polyP biosynthesis, Ppk1 (Table 1).

**Figure 2:**
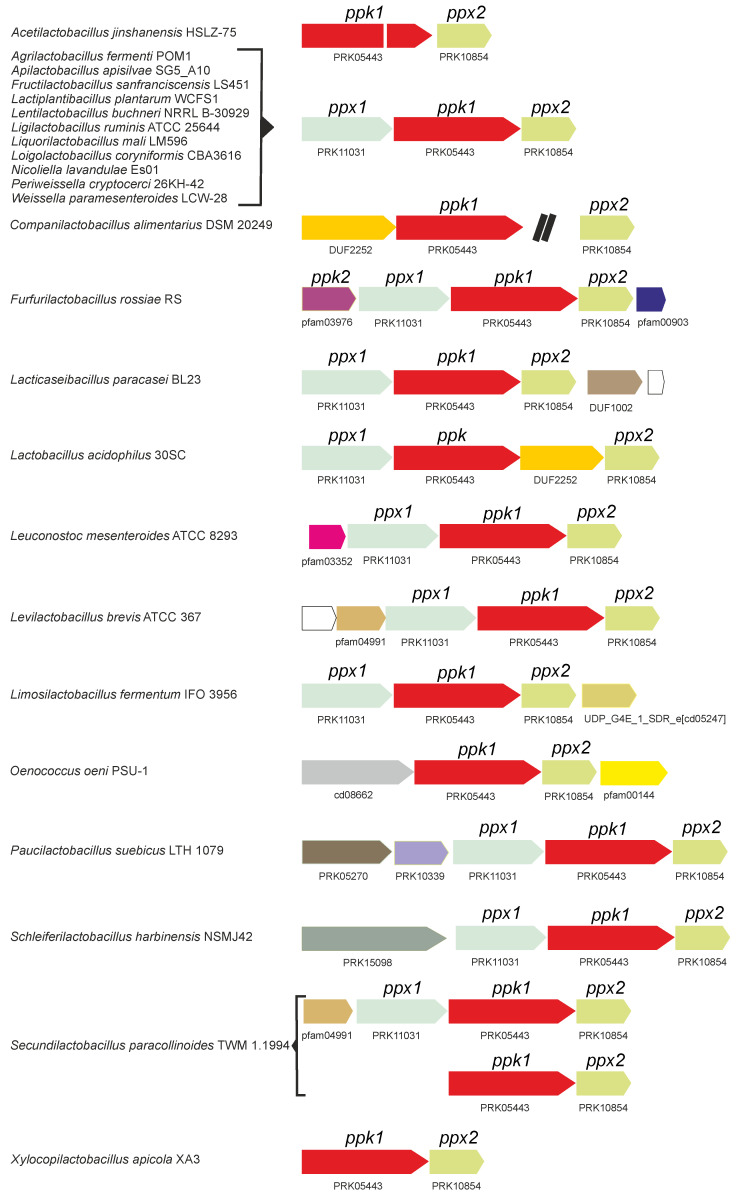
PolyP metabolic gene clusters identified in representative sequenced genomes of bacterial species within the *Lactobacillaceae* family. The presence of *ppk*, *ppx1*, and *ppx2* homologs, along with flanking genes in different strains and species, is depicted. Only genes presumably constituting operons together with *ppk1*, *ppx1*, or *ppx2* are represented. Homologous genes are indicated by matching colors. A double black bar represents an intervening genomic region that is not shown to scale.

Analysis of the genetic organization of polyP genes in *Lactobacillaceae* revealed that a gene cluster with the gene order *ppx1*-*ppk1*-*ppx2* is the most common gene arrangement in *Lactobacillaceae* species [34] (Figure 2). Additional genes may be present in some genera alongside this core cluster, although there is no evidence of their involvement on polyP metabolism. Notably, *ppx1* is absent in some genera such as *Acetilactobacillus*, *Companilactobacillus*, *Oenococcus*, and *Xylocopilactobacillus*, whereas *Secundilactobacillus* encodes two copies of *ppk1* and *ppx2* (Figure 2). In contrast, *ppk2* genes are usually monocistronic and located apart from the other polyP metabolic genes, with the exception of *Furfurilactobacillus*, where all genes are located within the same cluster (Figure 2).

### 2.1. Polyphosphate Kinases

Ppk1 is one of the most-studied enzymes in polyP metabolism. Ppk1 was first characterized in *Escherichia coli* [36,37,38] and subsequently in other bacteria such as *Mycobacterium smegmatis* [39], *Arthrobacter atrocyaneus* [40], *Propionibacterium shermanii* [41,42], and *Neisseria meningitidis* [43], among others. These enzymes transfer a terminal phosphate group to the polyP chain using ATP, leading to the progressive synthesis of an elongated polyP chain [4]. The determination of the structure of *E. coli* Ppk1 revealed that it forms a dimer, where the active site is located in a tunnel that penetrates the center of each monomer with an ATP-binding pocket on one side and conserved positively charged residues along the tunnel which possibly interact with the polyP chain during elongation [44].

**Figure 3:**
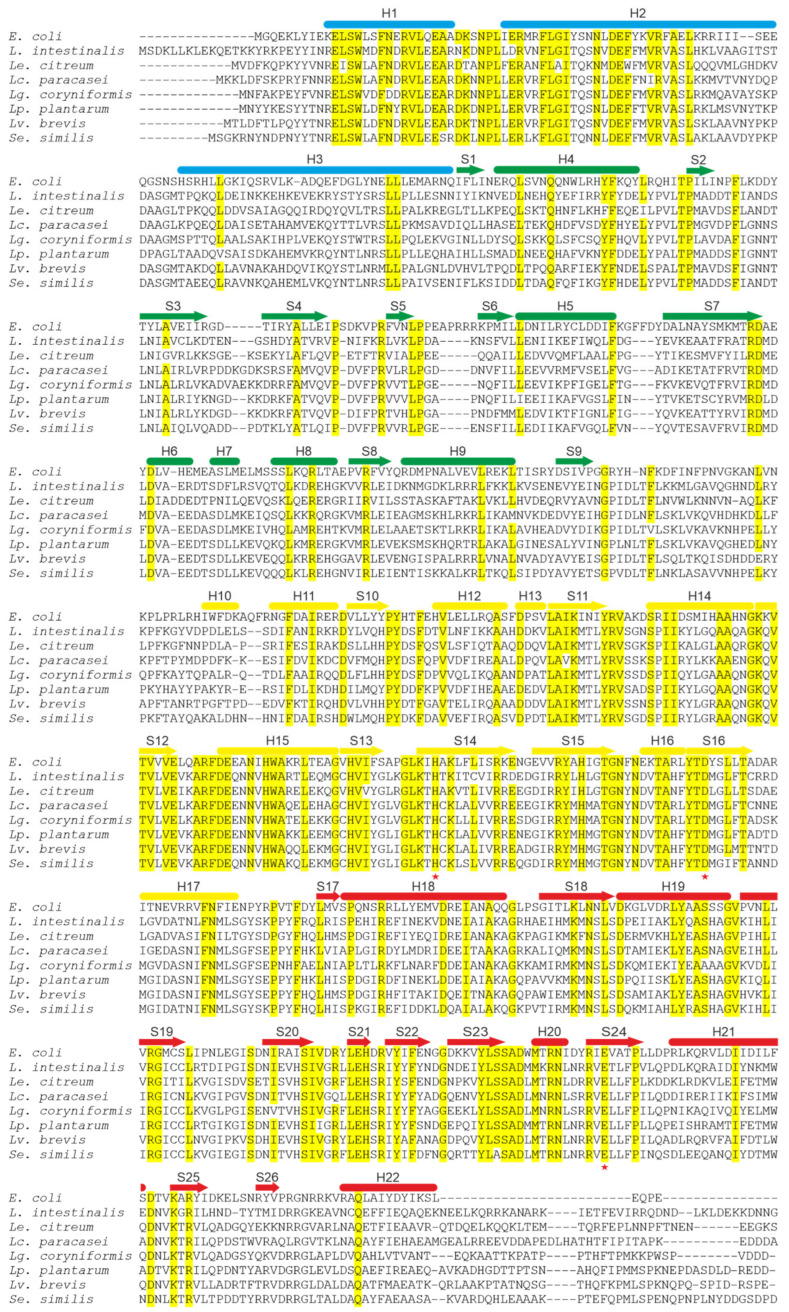
Sequence alignment of polyphosphate kinases (Ppk1) from representative *Lactobacillaceae* species and *Escherichia coli K12 (NP_416996.1)*. *Lactobacillaceae* sequences comprise *Lactobacillus intestinalis* (WP_304402131.1), *Leuconostoc citreum* (WP_349532772.1), *Lc. paracasei* BL23 (CAQ67833.1), *Loigolactobacillus coryniformis* (WP_146990929.1), *Lp. plantarum* WCFS1 (CCC78296.1), *Lv. brevis* (WP_141373826.1), and *Secundilactobacillus similis* (WP_057152190.1). Identical or highly conserved residues are indicated by yellow shading. Secondary structure elements derived from the *E. coli* Ppk1 structure as described by Zhu et al. [44] are shown above the sequences: α-helices are depicted as bars and β-strands as arrows. The colors of these elements correspond to the four structural domains of *E. coli* Ppk1. The phosphorylated residue H435 and the residues involved in autophosphorylation (D470 and E623) are marked with red stars.

In LAB, the presence of *ppk1* in the genome is associated with the polyP synthesis. Alcántara et al. [34] reported that 18 out of 34 lactobacillus strains accumulated polyP, and this accumulation correlated with the presence of *ppk1* genes in their genomes. A subsequent study that tested polyP accumulation in different strains of *Lactobacillus* and *Enterococcus* detected no polyP accumulation in *Enterococcus* strains lacking *ppk1* homologs [32]. However, in both studies, polyP accumulation varied widely even among different strains of the same species. This variability remains unexplained, as the regulation of polyP synthesis in LAB is poorly understood. In addition to differences in Ppk biosynthetic activity, variations in Pi uptake and polyP degradation may also contribute to this effect [32,34]. A dependence of polyP synthesis on Pi concentration has been observed in several LAB [34,45]. Although no LAB Ppk1 has been structurally or biochemically characterized in detail, a comparison of *Lactobacillaceae* Ppk1 sequences with that of *E. coli* reveals that critical residues for Ppk1 catalytic activity are conserved in *Lactobacillaceae* Ppks, suggesting that their structures possibly resemble that of *E. coli* Ppk1 (Figure 3). Furthermore, inactivation of the *ppk1* gene in *Lc. paracasei* BL23 abolished polyP synthesis in this organism [34] and the in vitro synthesis of polyP by *Lc. paracasei* Ppk1 cloned and expressed in *E. coli* has been demonstrated [35].

**Figure 4:**
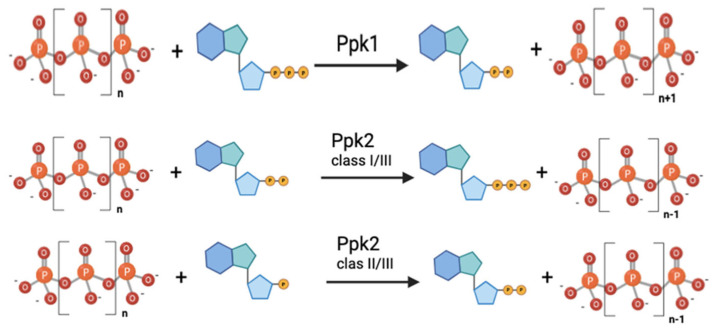
Enzymatic reactions carried out by the different polyphosphate kinases.

The discovery of Ppk2 originated from the observation that a *Pseudomonas aeruginosa* mutant defective in Ppk1 still produced up to 20% of the wild-type polyP levels [24,46]. This suggested that another enzyme might be responsible for additional polyphosphate kinase activity [46]. Subsequently, Ppk2 was identified as the enzyme responsible for synthesizing guanosine triphosphate (GTP) from guanosine diphosphate (GDP) and polyP in *P. aeruginosa* [47]. As noted above, Ppk2 proteins share no significant sequence similarity with Ppk1, and structural analyses have shown that they adopt different folds [24]. Biochemical analyses have revealed that *P. aeruginosa* Ppk2 exhibits a 75-fold preference for polyP degradation over synthesis [24,47]. Consequently, Ppk2 enzymes are more efficient in ATP/GTP synthesis due to their preference for nucleoside phosphorylation in the reversible reaction. Therefore, while Ppk1 acts preferentially in ATP-dependent polyP synthesis, Ppk2 is more efficient in synthesizing ATP/GTP from polyP (Figure 4).

**Figure 5:**
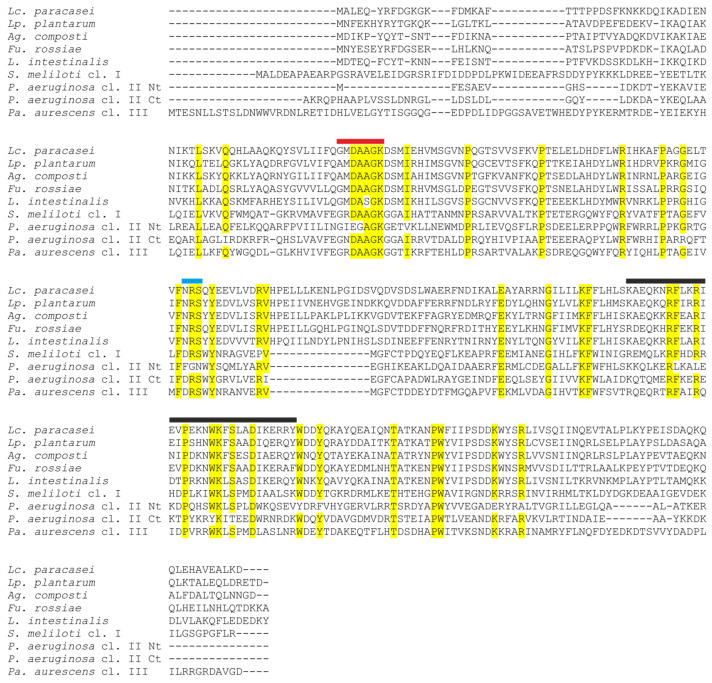
Sequence alignment of selected *Lactobacillaceae* species and representative sequences of the three subfamilies of polyphosphate kinase 2 proteins (Ppk2). Identical or highly conserved residues are indicated by yellow shading. The Walker A motif is designated by a red bar, the Walker B motif is indicated by a green bar, and the lid module is indicated by a black bar, as identified by Nocek, et al. [50]. *Lactobacillaceae* sequences comprise *Lc. paracasei* BL23 (CAQ66314.1), *Lp. plantarum* WCFS1 (WP_003642687.1), *Agrilactobacillus composti* DSM 18527 (KRM35117.1), *Furfurilactobacillus rossiae* DSM 15814 (KRL53865.1), and *L. intestinalis* DSM 6629 (KRM32733.1). *Sinorhizobium meliloti* 1021 (WP_010968631.1) represents class I Ppk2, *Pseudomonas aeruginosa* PAO1 (NP_252145.1) represents class II, and *Paenarthrobacter aurescens* TC1 (WP_261609683.1) represents class III. The two domains of *P. aeruginosa* class II Ppk2 are aligned separately and indicated as Nt and Ct, respectively.

Ppk2 is a member of the P-loop-containing kinases, which are characterized by the presence of two conserved sequence motifs (Walker A and Walker B) and a lid module. Ppk2 proteins possess deviant Walker A and B motifs compared to other P-loop kinases [48]. Based on phylogenetic, biochemical, and structural data, Ppk2 enzymes can be further classified into three subfamilies according to their nucleoside phosphate substrate preference: Class I phosphorylates nucleoside diphosphates, Class II phosphorylates nucleoside monophosphates, and Class III can phosphorylate both nucleoside mono- or diphosphates [49]. Although no *Lactobacillaceae* Ppk2 protein has been biochemically characterized, phylogenetic analysis places them in Class III [49], and the alignment of *Lactobacillaceae* Ppk2 sequences also shows conservation of the critical catalytic domains (Figure 5).

### 2.2. Exopolyphosphatases

Exopolyphosphatases (Ppx) progressively hydrolyze and release terminal Pi from linear polyP chains containing three or more phosphoanhydride bonds. In initial investigations in *E. coli*, a gene encoding exopolyphosphatase (*ppx*) was identified adjacent to the gene encoding Ppk1 (*ppk*). These two genes constitute a polycistronic operon in which transcription of the *ppx* gene is regulated by the *ppk* promoters [51]. Elucidation of the structure of *E. coli* Ppx revealed that the protein is composed of four domains and forms a dimer with a deep canyon at the dimer interface [52]. This canyon, lined with numerous basic residues, opens to the active site and is postulated to be the polyP binding site [52]. The active site region contains glycine-rich phosphate-binding loops named P-loops (distinct from the P-loops previously described in kinases). Domains I and II, which form the putative Ppx active site at their interface, share structural similarities with proteins possessing a ribonuclease-H-like fold. Domain III shows structural similarity to the N-terminal HD domain of the (p)ppGpp synthetase SpoT, whereas domain IV has structural counterparts in cold-shock-associated RNA-binding proteins [52]. *E. coli* Ppx is homologous to GppA and shares the same structural arrangement [53]. GppA regulates the pppGpp/ppGpp ratio in cells by converting pppGpp into ppGpp [54,55]. Despite their structural similarities, Ppx preferentially acts on polyP, whereas GppA targets pppGpp. This substrate specificity is attributed to conformational differences in their active sites, whereby Ppx adopts a closed conformation that hinders pppGpp binding, whereas GppA adopts an open conformation that facilitates it [56]. Other Ppx proteins, such as that encoded by *Aquifex aeolicus*, possibly act on both substrates, as it possess an active site with an open conformation [52,56]. *A. aeolicus* Ppx is monomeric and lacks the C-terminal domains III and IV [57].

**Figure 6:**
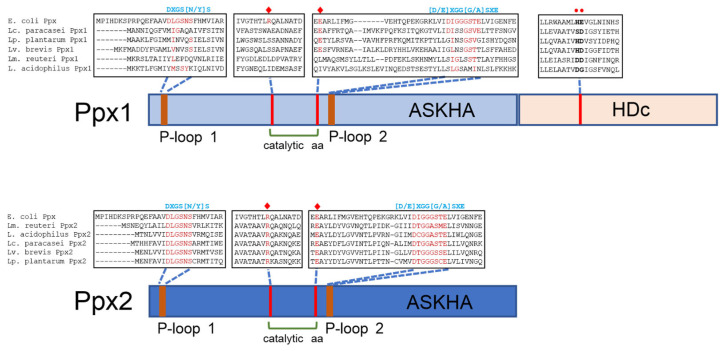
Schematic representation of Ppx1 and Ppx2. The presence of different protein domains (ASKHA and HDc) is depicted, together with relevant portions of multiple sequence alignments of Ppx1 from *Lc. paracasei* (WP_003606084.1), *Lv. brevis* (AYM03703.1), *Lp. plantarum* (WP_103851489.1), *Limosilactobacillus reuteri* (MCH5356744.1), and *Lactobacillus acidophilus* (MCT3601606.1) and from Ppx2 from *Lc. paracasei* (CAQ67832.1), *Lv. brevis* (WP_135367400.1), *Lp. plantarum* (WP_003641094.1), *Lm. reuteri* (KRK50994.1), and *L. acidophilus* (AZN76677.1), compared with Ppx from *E. coli* (WP_001121363.1). The consensus sequences of the conserved P-loop 1 and P-loop 2 in the GppA/Ppx family are shown in blue above the alignments. The catalytic amino acids arginine and glutamic acid are marked with red diamonds. The amino acids characteristic of the HDc domain in C-terminal Ppx1 are marked with red circles. Reproduced with permission from Corrales et al. [35].

As noted above (see Figure 2), members of the *Lactobacillaceae* family typically encode two Ppx proteins: Ppx1, which possesses the four domains present in *E. coli* Ppx, and Ppx2, which contains only the two N-terminal catalytic domains, similar to *A. aeolicus* Ppx. However, available evidence suggests that Ppx1 in *Lactobacillaceae* is not involved in polyP hydrolysis, unlike all other characterized Ppx1 homologs. It was observed that *Lc. paracasei* BL23, harboring an in-frame deletion of *ppx1* was unable to accumulate polyP. Complementation of *ppx1 in trans* restored polyP synthesis [35]. These results indicate not only that Ppx1 does not participate in polyP hydrolysis, but also that it is essential for polyP synthesis in *Lc. paracasei*. Inspection of the amino acid sequence of Ppx1 proteins from *Lactobacillaceae* revealed amino acid substitutions at key residues thought to be critical for polyphosphatase catalytic activity (Figure 6), particularly within conserved P-loops and catalytic residues [35].

**Figure 7:**
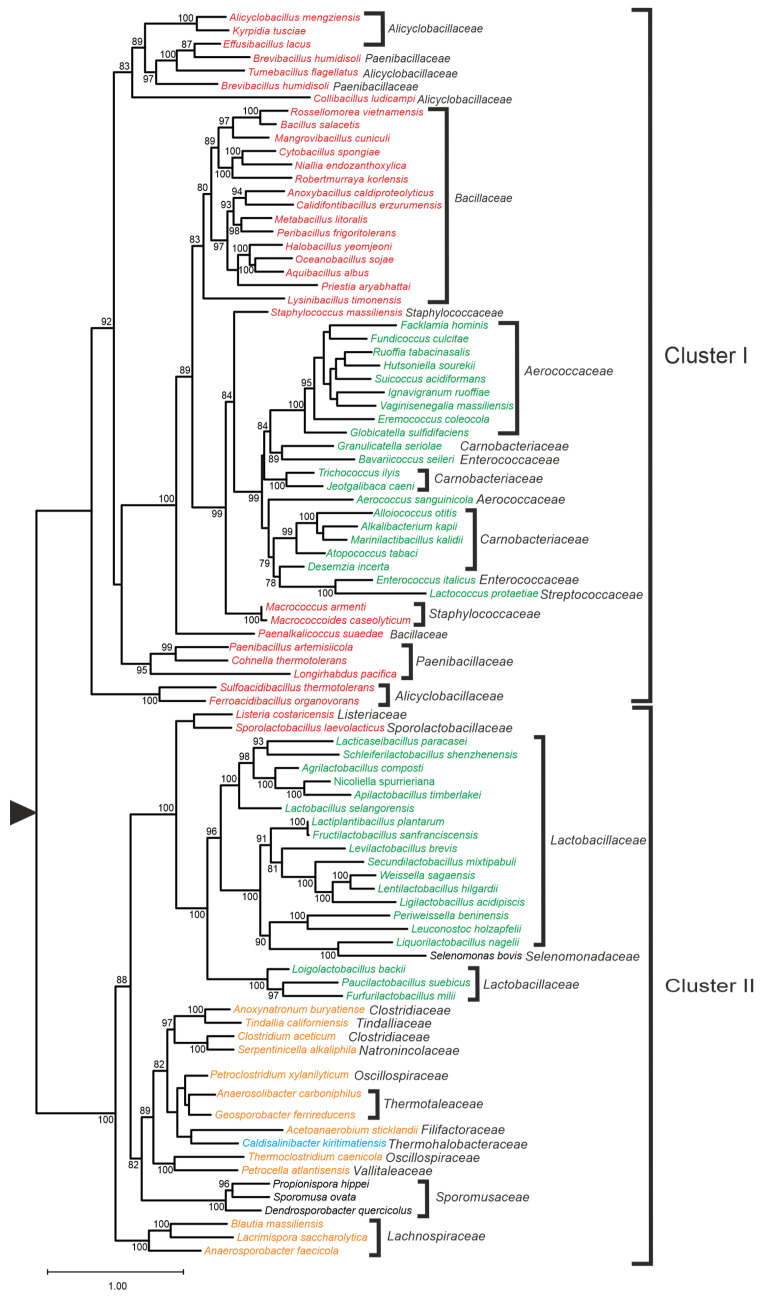
Maximum likelihood phylogenetic tree of Ppx1 proteins of selected species of the phylum *Bacillota*. Support values are given for those nodes with support higher than 75%. The tree has been arbitrarily rooted for ease of visualization. Colors: black: class *Negativicutes*; brown: class *Clostridia*; blue: class *Tissierellia*; green: order *Lactobacillales*; and red: order *Bacillales*. Reproduced with permission from Corrales et al. [35].

A phylogenetic analysis of Ppx1 homologs from taxa within the phylum *Bacillota* revealed two major clusters (Figure 7). Cluster I encompasses Ppx1 proteins from taxa in the orders *Bacillales* and *Lactobacillales*, excluding those from the family *Lactobacillaceae*. Cluster II primarily comprises proteins from *Lactobacillaceae*, as well as from the classes *Clostridia* and *Negativicutes*. Inspection of the sequences revealed that Cluster I Ppx1 proteins retain conserved motifs and residues involved in polyphosphatase activity, whereas Cluster II proteins exhibit changes in some or most of these residues [35]. These findings suggest that Cluster II Ppx1 proteins lack polyphosphatase activity and might instead function as activators of their Ppk1 counterparts, as described in *Lc. paracasei* BL23. The mechanism by which Ppx1 regulates Ppk1 activity remains to be elucidated. Notwithstanding, it is worth noting that some species, such as *Oenococcus oeni* and *Xylocopilactobacillus apicola*, encode only the *ppx2* gene (Figure 2). Unfortunately, evidence regarding their ability to synthesize polyP is lacking.

On the other hand, the purified Ppx2 enzyme from *Lc. paracasei* BL23 has been shown to exhibit in vitro exopolyphosphatase activity and displays full conservation of P-loops 1 and 2, along with other key catalytic amino acid residues (Figure 6) [35]. These findings suggest that Ppx2 is an active polyphosphatase in *Lactobacillaceae*. However, additional polyP-consuming activities must be present, as inactivation of *ppx2* in *Lc. paracasei* BL23 did not affect polyP accumulation or degradation in this organism [35].

## 3. Physiological Roles of Polyphosphate in Lactic Acid Bacteria

**Figure 8:**
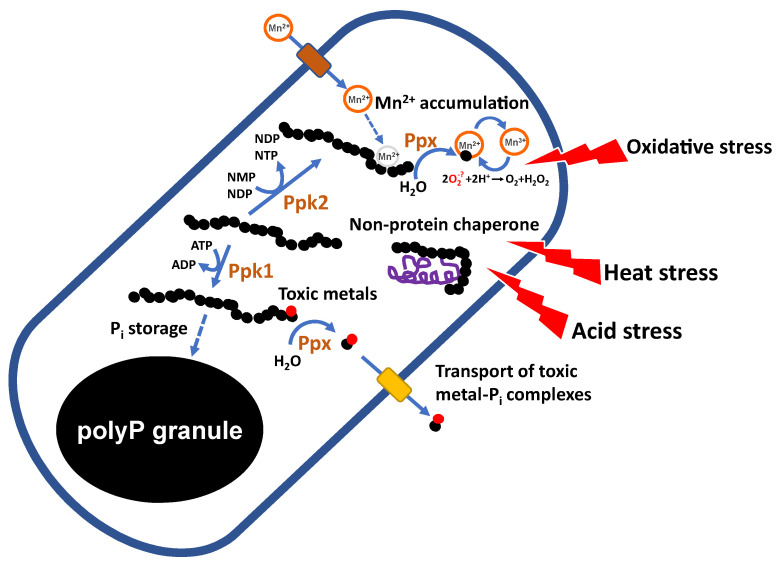
Proposed roles of polyP in the physiology of LAB. PolyP may play roles in detoxification of metals, responses to heat and acid stresses, response to oxidative stress, by participating in the accumulation of Mn^2+^, and in the synthesis of nucleotides. NMP, nucleotide monophosphate; NDP, nucleotide diphosphate; NTP, nucleotide triphosphate.

PolyP is involved in a wide range of physiological processes across all domains of life [2,58,59,60], with its specific functions depending on the organism and cellular context. In bacteria, polyP participates in several processes, including: (i) serving as a structural component of lipid membranes [61] and contributing to the formation of non-protein Ca^2+^ channels [62]; (ii) supporting resistance to various stress conditions [63,64]; (iii) acting as an ATP substitute, an energy source, and a Pi reservoir [65,66,67]; (iv) contributing to nucleoid and chromosomal organization as well as gene regulation [2]; (v) regulating cell division and morphology [68]; and (vi) enhancing the virulence of certain pathogens [69,70]. Despite extensive research on these roles in various bacterial taxa, the involvement of polyP in the physiology of LAB remains comparatively underexplored (Figure 8).

The relationship between polyP and the stress response is the most extensively studied aspect of polyP metabolism in LAB and has emerged as a key factor in their microbial physiology. As a polyanion, PolyP is a strong chelator of metal cations [66], a property proposed to increase the tolerance of polyP-producing microorganisms to toxic metals. Several studies have shown that both polyP synthesis and degradation are required for this detoxifying function. A proposed model suggests that toxic metals are initially sequestered by polyP which is then degraded by polyphosphatases. The resulting metal-Pi complexes are subsequently exported via Pi transporters [71]. Whether LAB use a similar mechanism remains to be elucidated.

In LAB, interactions with heavy metals have been suggested to occur primarily at the bacterial surface through binding to cell wall components [72]. Intracellular interactions between polyP and metals in LAB has only been described for Mn^2+^. *Lp. plantarum* has been shown to accumulate over 20 mM of intracellular Mn^2+^ [30,73] as a mechanism of defense against oxidative stress. While the exact protective role of polyP under oxidative conditions is not yet fully understood, several mechanisms of action have been proposed. For example, its activity against superoxide ion (O^2−^) can be explained by its ability to coordinate cations such as Fe^3+^ or Mn^2+^, forming complexes that facilitate O^2−^ dismutation [66]. In this model, Mn^2+^ is chelated by polyP, forming a Mn^2+^-polyP complex, which is then hydrolyzed by exopolyphosphatases to generate MnHPO_4_, a compound capable of detoxifying O^2−^.

PolyP may also inhibit the Fenton reaction, which generates highly reactive hydroxyl radicals, by chelating cations such as Fe^2+^ or Cu^2+^ and stabilizing the Fe^3+^ intermediates [63,66]. In, *Lp. plantarum*, polyP-complexed Mn^2+^ also appears to contribute directly to O^2−^ scavenging [74]. Notably, Pi limitation reduces Mn accumulation in *Lp. plantarum* ATCC 14917 [75], and, conversely, Mn-depleted conditions inhibit polyP synthesis in both *Lp. plantarum* [75] and *Lacticaseibacillus rhamnosus* ATCC 7469 [76]. These findings suggest a regulatory mechanism coordinating intracellular Mn and Pi accumulation.

Mutants of *Lc. paracasei* unable to produce polyP due to disruption of the *ppk1* gene exhibited increased sensitivity to osmotic (NaCl), acidic (pH 4), and oxidative (plumbagin-induced) stresses compared to the wild-type strain [34]. In contrast, inactivation of *ppk1* in *Lp. plantarum* did not affect sensitivity to osmotic or acidic stress. However, *ppk1* mutants of both *Lp. plantarum* and *Lc. paracasei* were more susceptible to both inorganic and organic mercury [72].

Heat stress is a relevant factor in industrial applications of LAB. In *Lc. rhamnosus* CRL1505, high content of polyP in the presence of inorganic salts (MnSO_4_, MgSO_4_, and Pi) was associated with increased survival under heat stress [33]. Similarly, *Lc. paracasei* BL23 grown in hyper-concentrated (30%) sweet whey formed intracellular polyP granules, which were absent under isotonic (5% sweet whey) conditions. This high-polyP condition also correlated with enhanced survival during spray drying, suggesting a role for polyP in thermotolerance [77]. Although the precise mechanisms behind polyP-mediated stress resistance in LAB remain unclear, several pathways may be involved, including links to oxidative stress resistance and the chaperone activity of polyP [64]. Notably, the potential role of polyP in transcriptional regulation during stress response has not been explored in LAB. In *E. coli*, the general stress response is controlled by the sigma factor σ38 (encoded by *rpoS*) whose transcription requires both the Ppk enzyme and polyP. Moreover, *rpoS*-deficient mutants fail to accumulate polyP under osmotic stress or nitrogen limitation [63,78,79]. However, LAB generally lack alternative sigma factors, implying that different, yet unidentified, regulatory mechanisms likely mediate the connection between polyP and stress responses in these organisms.

Studies using *E. coli* strains deficient in the *ppk1* gene revealed increased sensitivity to hypochlorous acid (HOCl) treatment compared to the wild-type strain. HOCl causes severe protein damage by oxidizing amino acids such as cysteine, methionine, and histidine, leading to protein unfolding and aggregation. PolyP has been shown to interact directly with unfolded proteins, preventing their aggregation and thereby mitigating HOCl-induced stress [64]. In contrast, inactivation of *ppk1* in *Limosilactobacillus reuteri* does not significantly impair resistance to HOCl-induced protein damage. Notably, a *ppk1* mutation in this species only reduces polyP levels by approximately 50%, suggesting that the Ppk2 enzyme also contributes to polyP synthesis [80]. However, mutation of *ppk2* in this bacterium leads to a strong defect in HOCl resistance [81], suggesting that ATP/GTP generation via Ppk2 activity may be critical for stress survival in this species.

## 4. Regulation of Polyphosphate Synthesis in Lactic Acid Bacteria

Despite intensive research into bacterial polyP synthesis in recent years, the mechanisms regulating its synthesis and degradation remain largely unknown. In lactobacilli, wide differences in polyP content have been reported, even between species sharing the same genetic organization of polyP metabolic genes [34,82]. These results suggest that differential regulation may occur in different lactobacilli species, although evidence available so far is insufficient to ascertain this point. Early models based on *E. coli* proposed that the alarmone (p)ppGpp inhibits Ppx exopolyphosphatase activity, thereby promoting polyP accumulation. However, these models have recently been questioned [83], and no evidence currently supports a role for (p)ppGpp in polyP accumulation in LAB. Similar to other bacteria, polyP levels in LAB rise during exponential growth and sharply increase at the onset of the stationary phase [32], a phenomenon likely triggered by signals related to nutritional downshift. PolyP concentrations in LAB growth supernatants are much lower than intracellular levels, but follow a similar temporal pattern. The role of polyP degradation during the stationary phase, whether mediated by Ppx or Ppk2, remains unknown. Additionally, growth under high Pi conditions significantly enhances polyP accumulation in LAB compared to growth under Pi-limited conditions [34,35]. Regulation of the transcription of polyP metabolism genes has been little studied in lactobacilli. Transcription of *ppk1*, *ppx1*, and *ppx2* has been observed in *Lc. rhamnosus* CRL1505 concomitant with polyP synthesis [84] and growth under high- or low-Pi conditions had only a moderate effect on *ppx/ppk* expression in *Lc. paracasei* BL23 [35]. Furthermore, no effect of the PhoP regulator, which controls transcription of Pi-regulated genes, has been evidenced for *ppk*, *ppx1*, or *ppx2* expression in this species [85]. The fact that the genes encoding polyP synthesizing and degrading enzymes are clustered in an operon in LAB and are jointly transcribed, probably excludes transcriptional regulation as the main control mechanism. However, different regulation may exist for *ppk2*, which is not located in *ppx1-ppk1-ppx2* gene clusters. In *Lactiplantibacillus paraplantarum* CRL1905, the enzyme Ppk2 was not detected in proteomic analyses when cells were cultivated in medium with high Pi, while it was present under low Pi conditions. This correlated with increased accumulation and persistence of polyP at the stationary phase under high Pi in this strain [45].

An *Lc. paracasei* mutant strain has been isolated that displayed an enhanced polyP production (100-fold increase compared to the parental strain) [32]. In this mutant strain, extracellularly located polyP also followed a similar trend. Unfortunately, the nature of the mutation(s) responsible for this enhanced production is not known. A mutant strain has also been characterized from *Lp. paraplantarum* KCCM11826P which consumed 76% more Pi from culture medium than its parental strain and produced more polyP. In this case, mutations in *tuf*, *dnaK*, and *groL* genes were found in the genome of this high polyP accumulating strain [86]. This likely links polyP accumulation to a mechanism triggered by some type of stress that resulted from defects in protein folding and/or translation elongation.

As already mentioned, in contrast to Ppx2, Ppx1 from LAB does not possess the characteristics of a true exopolyphosphatase [35]. This observation, combined with the fact that deletion of the gene encoding this enzyme in *Lc. paracasei* BL23 completely abolishes polyP synthesis, strongly suggests that polyP accumulation in LAB is controlled through signal integration affecting Ppk1 and/or Ppx2 enzymatic activity, with Ppx1 playing a regulatory role. However, the specific nature of this putative regulatory mechanism remains unclear.

## 5. Functional Properties of Probiotic-Derived Polyphosphate: Current Evidence

**Figure 9:**
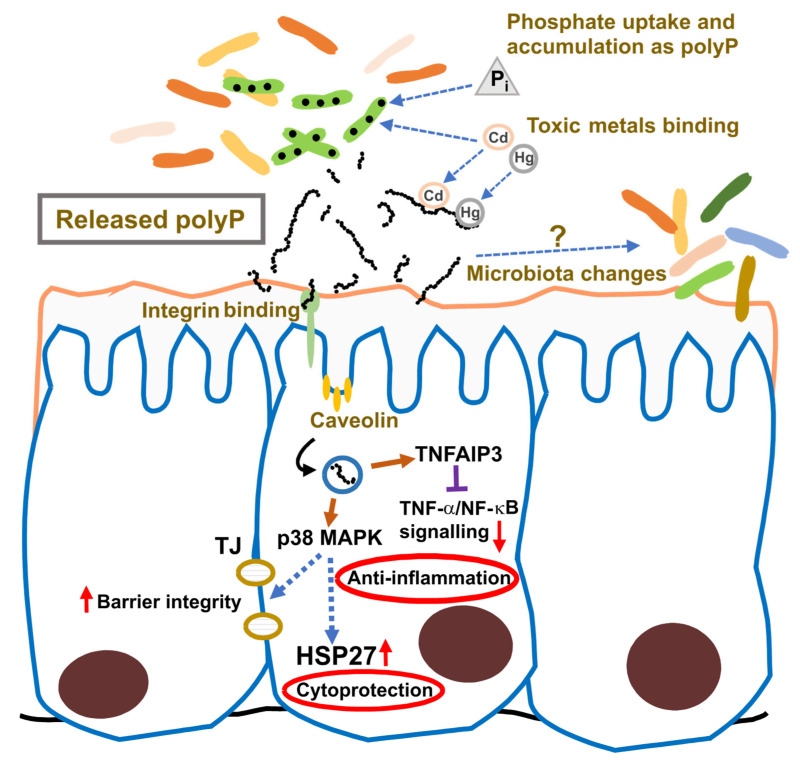
Proposed roles of probiotic-derived polyP in the gastrointestinal mucosa. The intestinal mucosa and associated microbiota is represented. PolyP released by intestinal probiotic bacteria, such as lactic acid bacteria, may have different beneficial functions by regulating certain anti-inflammatory and cytoprotective pathways, regulating toxic metals and phosphate bioavailability and inducing changes in the microbiota composition. TJ, tight junctions. Red up arrows indicate increased activity, and red down arrows indicate decreased activity.

In prokaryotes, polyP is primarily found intracellularly. In contrast, higher eukaryotes, including humans, produce polyP both intra- and extracellularly, where it is involved in a wide range of physiological processes. In humans, polyP plays roles in diverse functions across different tissues, such as blood coagulation, osteogenesis and chondrogenesis, neurotransmission, regulation of mitochondrial energetics, inflammation, and the response to infections, among others [58,59,87]. As previously noted, polyP produced by LAB has been identified as a functional molecule present in the supernatants of probiotic cultures. This extracellular polyP contributes to the maintenance of intestinal homeostasis and has recently attracted interest for its potential therapeutic applications. The capacity of certain LAB to accumulate and to excrete polyP to some extent may influence host inflammatory pathways and Pi balance (Figure 9). Understanding the underlying mechanisms of these effects could lead to novel strategies for managing inflammatory diseases and disorders associated with Pi imbalance, among others.

Early research showed that *Lv. brevis* SBC8803 induces cytoprotective heat-shock proteins in the small intestine and protects against intestinal injury in a mouse model of acute colitis [88]. The responsible factor, identified in the bacterial culture supernatant, was polyP [19]. This probiotic-derived polyP replicated the beneficial effects of the SBC8803 strain in Caco-2/BBE cells, inducing HSP27 and protecting cells against oxidative damage caused by H_2_O_2_ or NH_2_Cl. In a murine colitis model, polyP suppressed the induction of the proinflammatory cytokines IL-1β and IL-6 and improved survival [19]. Mechanistic analyses indicated that polyP protects intestinal barrier function via the integrin-p38 MAPK signaling pathway with no apparent action on other MAPK pathways such as ERK, JNK, or Akt. Analyses using integrin β1 antagonists showed that the protective effect of polyP was abolished, and coimmunoprecipitation assays with ^32^P-labeled polyP demonstrated that polyP binds to integrin β1. These results demonstrated that polyP interacts with integrin β1, leading to the activation of p38 MAPK [19]. Unlike other host–microbe interaction factors, polyP signaling is not mediated by pattern recognition receptor (PRR) pathways. Instead, it is internalized through the integrin–caveolin-mediated endocytic pathway and induces TNFAIP3 (tumor necrosis factor alpha-induced protein 3) expression in Caco-2/BBE cells. This provides a mechanistic basis for its anti-inflammatory properties through inhibition of TNF-α/NF-κB signaling and enhancement of intestinal barrier function [89].

Further studies in mouse models of disease support the anti-inflammatory and barrier-protective properties of LAB-derived polyP. In chronic colitis models (TNBS- and DSS-induced), polyP prevented fibrosis by suppressing expression of collagen types I and IV, along with proinflammatory cytokines IL-1β, TNF-α, and IFN-γ [90]. This effect was attributed to the downregulation of TGF-β1 expression in Caco-2/BBE epithelial cells, but not in THP-1 macrophages. Notably, polyP did not reduce collagen expression in TGF-β1-stimulated CDD-18 fibroblasts, suggesting that epithelial cells are the primary targets of polyP action [90]. In addition, polyP isolated from *Lp. plantarum* (average length of 250 Pi residues) inhibited lipopolysaccharide-stimulated M1 polarization of RAW264.7 macrophages, providing another alternative anti-inflammatory mechanism [91]. When IEC-18 cells were incubated with platelets pretreated with probiotic-derived polyP, this led to activation of the ERK, Akt, JNK, and p38 pathways. These platelets released unidentified factors that activated the Raf–MEK signaling cascade upstream of ERK, promoting epithelial wound healing in vitro [92]. This response was linked to the beneficial effects of polyP on DSS-induced colitis in mice, where platelet accumulation at the intestinal epithelium was observed [92]. Recent evidence has also shown an additional link between polyP and blood coagulation. PolyP derived from *Staphylococcus aureus* activates factor XII which in turn induces fibrin production [93]. The authors suggest that this interaction leads to abcess formation and thus to limitation of bacterial infection.

The effects of LAB-derived polyP have also been explored beyond the intestinal tract. Oral administration of polyP from *Lv. brevis* (1 mg/kg body weight) to mice alleviated acute pancreatitis induced by intraperitoneal injection of cerulein. Treatment with polyP suppressed histological signs of inflammation in the pancreas, diminished expression of IL-6 and the monocyte chemoattractant protein-1 (MCP-1/CCL2) in the pancreas, and lowered serum levels of amylase and lipase [94]. Additionally, polyP administration modified the composition of the intestinal microbiota, decreasing the abundance of potentially virulent bacteria such as *Desulfovibrio*, while increasing levels of potentially beneficial bacteria, such as *Alistipes* and *Candidatus Saccharimonas*. It remains unclear whether these microbiota changes result from a direct effect of polyP or are secondary to polyP-induced modifications of the intestinal epithelium.

Although most studies on polyP functionality have utilized polyP isolated from LAB cultures or enzymatically synthesized polyP (via Ppk), other investigations have tested functionality directly using LAB culture supernatants or whole cells. Growth supernatants from *Lp. plantarum* WCFS1 and *Lp. plantarum* Lpp+ induced HSP27 expression in Caco-2 cultures, whereas supernatants from their respective *ppk1* mutants failed to elicit this response [72]. Similarly, supernatants from *Lm. reuteri* SBC8803 cultures induced apoptosis in the human colon carcinoma SW620 cell line, but had no such effect on primary epithelial cells derived from normal small intestine. This antitumor effect was abolished when polyP present in these supernatants was hydrolyzed by Ppx treatment [95]. In this case, the antitumor effect of polyP was associated with activation of the ERK pathway [95]. The polyP-accumulating strain *Lc. rhamnosus* CRL1505 was also studied in a murine model of acute respiratory inflammation induced by lipopolysaccharide [84]. Intracellular extracts rich in polyP from this strain normalized serum levels of several pro-inflammatory cytokines (IL-17, IL-6, IL-2, IL-4, IFN-γ) in nasally-treated mice, suggesting a protective effect against respiratory tract inflammation [84]. These findings support the potential of LAB-derived polyP as an innovative biotherapeutic agent for inflammatory diseases beyond the gastrointestinal tract. However, some studies have pointed to possible negative effects for polyP-accumulating LAB. Certain LAB species present in the oral cavity that are capable of accumulating polyP may have negative health impacts by facilitating tooth demineralization. This was evidenced by experiments with *Lc. rhamnosus*, a bacterium found in carious lesions, which showed that Pi draining for polyP synthesis may create physicochemical conditions that favor tooth dissolution [76,96].

In addition to their proposed role in maintaining host homeostasis via modulation of signaling pathways, recent studies have highlighted further biotechnological and health-related applications of polyP-producing LAB. One promising avenue is the treatment of hyperphosphatemia in patients with chronic kidney disease (CKD) by reducing intestinal phosphorus absorption. In a rat model of CKD, supplementation with Lp. *paraplantarum* KCCM 11826P significantly lowered serum Pi levels [97]. This attenuation is attributed to the remarkable capacity of this strain to absorb Pi and store it as polyP, suggesting a potential alternative to conventional Pi binders for phosphorus control in CKD patients [97]. Additional beneficial properties have also been ascribed to lactobacilli-derived polyP. Due to its high negative charge density, polyP is hypothesized to chelate toxic metal cations such as Hg^2+^ and Cd^2+^. Probiotic strains generally demonstrate a high capacity for binding such cations, potentially decreasing their bioavailability and toxicity in the gastrointestinal tract [98,99]. However, studies using *ppk1* mutants deficient in polyP synthesis of *Lc. paracasei* and *Lp. plantarum* revealed no reduction in their metal-binding capacity despite increased sensitivity to mercury exposure [72]. These findings challenge the presumed direct role of polyP in heavy metal sequestration in LAB.

## 6. Future Prospects

Overall, polyP has emerged as a key factor in the functionality of probiotic LAB. Its roles in microbial physiology, particularly in stress resistance, cellular homeostasis, and adaptation to adverse environments, are increasingly recognized as central to LAB survival in the gastrointestinal tract and their ability to confer health benefits. Nonetheless, further research is essential to optimize polyP metabolism for industrial and probiotic applications. Additionally, the potential risks associated with polyP-accumulating LAB, particularly in the oral environment, need to be evaluated. In this sense, the use of delivery technologies, such as encapsulation, that reduce the risk of colonization in the oral cavity, should be considered. The capacity to synthesize polyP has been described as a virulence factor in some pathogens. Furthermore, the addition of exogenous polyP reversed the detrimental effects of the inactivation of *ppk1* in *Acinetobacter baumanni* [70]. This result emphasizes the need to investigate potential detrimental interactions between pathogens and polyP-accumulating LAB. Utilization of probiotic poly-P accumulating LAB is not currently subject to specific regulation. However, if safety issues arise, this will become an important consideration for the food industry. Many polyP-accumulating LAB strains are already commercialized as probiotics, but in most cases, polyP levels have not been taken into account during their production and delivery. These processes may need to be revised if high polyP levels are desired, along with their potential effects on various food properties, such as sensory characteristics and stability during storage, particularly when these strains are incorporated into food products.

Notably, the pathways governing polyP synthesis and degradation in LAB appear to diverge from those described in classical bacterial models, underscoring the need for targeted studies in this group. A deeper understanding of the regulatory mechanisms controlling polyP accumulation, particularly under stress conditions, is necessary to enhance its production and functional availability. Specifically, elucidating whether polyP reaches the extracellular environment through active secretion or as a result of cell lysis is essential, as this determines its availability to interact with host target cells. Finally, since polyP chain length critically influences its biological activity [87], identifying LAB strains that preferentially synthesize polyP of specific average lengths represents a promising area for strain selection and engineering. In this respect, challenges such as the cost and scalability of the processes required to obtain high polyP-accumulating or -excreting LAB must also be considered. Advancing our knowledge in these areas will be instrumental in the rational development of LAB strains with enhanced functionalities tailored for a range of biotechnological and therapeutic applications.

## Figures and Tables

**Table 1:** Presence of polyP metabolic genes in family *Lactobacillaceae*.

Genus	*ppk1*	*ppk2*	*ppx1*	*ppx2*
*Acetilactobacillus*	+	−	−	+
*Agrilactobacillus*	+	±	+	+
*Amylolactobacillus*	−	−	−	−
*Apilactobacillus*	±	−	±	±
*Bombilactobacillus*	−	−	−	±
*Companilactobacillus*	+	−	−	+
*Convivina*	−	−	−	+
*Dellaglioa*	−	−	−	+
*Eupransor*	+	−	−	+
*Fructilactobacillus*	+	−	±	+
*Fructobacillus*	±	−	−	+
*Furfurilactobacillus*	+	+	+	+
*Holzapfeliella*	−	−	−	−
*Lacticaseibacillus*	+	±	+	+
*Lactiplantibacillus*	+	+	+	+
*Lactobacillus*	±	±	±	±
*Lapidilactobacillus*	−	−	−	−
*Latilactobacillus*	−	−	−	−
*Lentilactobacillus*	±	±	±	+
*Leuconostoc*	+	−	+	+
*Levilactobacillus*	+	+	+	+
*Ligilactobacillus*	±	−	±	±
*Limosilactobacillus*	+	+	+	+
*Liquorilactobacillus*	+	±	+	+
*Loigolactobacillus*	±	±	±	±
*Nicoliella*	+	−	+	+
*Oenococcus*	+	−	−	+
*Paucilactobacillus*	±	−	±	+
*Pediococcus*	−	−	−	+
*Periweissella*	+	−	+	+
*Philodulcilactobacillus*	+	−	−	+
*Schleiferilactobacillus*	+	+	+	+
*Secundilactobacillus*	±	−	±	+
*Weissella*	±	−	±	+
*Xylocopilactobacillus*	+	−	−	+

Note: Genes were detected by blastp using as query sequences Ppk1 (CAQ67833.1), Ppk2 (CAQ66314.1), Ppx1 (CAQ67834.1), and Ppx2 (CAQ67832.1) from *Lc. paracasei* BL23 against the nr database using the NIH blast server. Sequences sharing the same conserved domains and over 90% sequence alignment coverage were considered homologous. + >90% species harbor the target gene; − 0%; ± <90%.

## Data Availability

No new data were created or analyzed in this study. Data sharing is not applicable to this article.

## Abbreviations

The following abbreviations are used in this manuscript:

DSS	Dextran Sodium Sulfate
ERK	Extracellular signal-Regulated Kinase
LAB	Lactic Acid Bacteria
MAPK	Mitogen-Activated Protein Kinase
PolyP	Polyphosphate
TNBS	2,4,6-trinitrobenzenesulfonic acid
